# Bacteriophage-Based Vaccines: A Potent Approach for Antigen Delivery

**DOI:** 10.3390/vaccines8030504

**Published:** 2020-09-04

**Authors:** Alejandro González-Mora, Jesús Hernández-Pérez, Hafiz M. N. Iqbal, Marco Rito-Palomares, Jorge Benavides

**Affiliations:** 1 Tecnologico de Monterrey, School of Engineering and Sciences, Ave. Eugenio Garza Sada 2501, Monterrey, N.L. 64849, Mexico; A00819537@itesm.mx (A.G.-M.); jhz.perez@tec.mx (J.H.-P.); hafiz.iqbal@tec.mx (H.M.N.I.); 2 Tecnologico de Monterrey, School of Medicine and Health Sciences, Ave. Morones Prieto 3000 Pte, Monterrey, N.L. 64710, Mexico; mrito@tec.mx

**Keywords:** bacteriophage, vaccines, phage display technology, immunological mechanism, antigen delivery

## Abstract

Vaccines are considered one of the most important bioproducts in medicine. Since the development of the smallpox vaccine in 1796, several types of vaccines for many diseases have been created. However, some vaccines have shown limitations as high cost and low immune responses. In that regard, bacteriophages have been proposed as an attractive alternative for the development of more cost-effective vaccines. Phage-displayed vaccines consists in the expression of antigens on the phage surface. This approach takes advantage of inherent properties of these particles such as their adjuvant capacity, economic production and high stability, among others. To date, three types of phage-based vaccines have been developed: phage-displayed, phage DNA and hybrid phage-DNA vaccines. Typically, phage display technology has been used for the identification of new and protective epitopes, mimotopes and antigens. In this context, phage particles represent a versatile, effective and promising alternative for the development of more effective vaccine delivery systems which should be highly exploited in the future. This review describes current advances in the development of bacteriophage-based vaccines, with special attention to vaccine delivery strategies. Moreover, the immunological aspects of phage-based vaccines, as well as the applications of phage display for vaccine development, are explored. Finally, important challenges and the future of phage-bases vaccines are discussed.

## 1. Introduction

The development of vaccines represents one of the greatest advances in medical fields which have saved a huge number of human and animal lives [1]. Nowadays, several research groups around the world have focused on the development of more effective, better, safer, inexpensive and with long-lasting immune response vaccines for medical applications [2,3,4]. Currently, different types of vaccines such as live-attenuated, inactivated, synthetic, among others have been successfully developed and tested for preventive purposes. Conventional vaccines are made of inactivated or attenuated microorganisms. Although significant improvements have been made regarding conventional vaccines, drawbacks as difficulties to culture microorganisms, low efficacies and potential risks related to pathogenic reversion or transmission to immunocompromised patients of these vaccines have been reported [4]. Therefore, the administration of specific and contaminant-free antigens has been proposed as a new strategy to overcome the limitations previously mentioned. Thus, novel vaccines based on recombinant peptides or proteins, synthetic peptides, dendritic-based and nucleic acid vaccines have been developed.

Recombinant vaccines have proved to be safe and easy to produce, leading to large amounts of this kind of vaccines to be currently in the market. However, some vaccines produced via recombinant DNA technology have shown to lack some of the immunogenic characteristics of the original target and tend to fail in the stimulation of the immune system [5]. Hence, adjuvant molecules are mixed with the vaccine to improve the immunogenic response. Although several adjuvant compounds have been developed and tested, their use remains questionable as the production cost of the vaccine increases [6]. 

Synthetic vaccines against pathogens such as HIV and *Plasmodium falciparum* have gained great attention in recent years [7]. However, the high production costs of synthetic molecules limit their commercial applications enormously, including veterinary vaccination programs in developing countries or regions where large quantities are needed [2]. Furthermore, it has been reported that the use of synthetic peptides, as well as naked DNA-based vaccines, have poor immunostimulatory effects [8,9]. Although novel vaccines are being designed to overcome the limitations of conventional vaccines, these approaches have presented several disadvantages. Thus, new and more reliable approaches for vaccine delivery have been proposed.

Bacteriophage-based vaccines are considered to become a potent alternative to overcome the limitations of classical vaccines. This approach takes advantage of bacteriophages’ inherent properties to improve the stability and immunogenicity of displayed antigens [10]. At the same time, this strategy benefits from the phage particles ability to stimulate both, cellular and humoral immunity [6,11]. The development of phage-based vaccines is possible due to the enhancement of molecular tools that allow the manipulation of phage genomes through the phage display technology and at the same time it is benefited by the improvement of microbiology, physiology, and immunology areas. This recent development of bacteriophages as vaccine delivery systems has opened a novel area for commercial growth [6]. Although several phage-based vaccines have been developed for human applications, veterinary approaches have gained more relevance in this market since the regulations are more flexible. In veterinary applications, the cost and efficacy of vaccines represent the main research area since the production of animals must be cost-effective [4]. In that regard, the recombinant bacteriophage technology represents one possible solution to surpass the limitations of current vaccines [2]. Besides, the increasing demand for novel vaccines for emerging pathogens can be addressed by the phage-vaccine approach.

The objective of this review is to describe current advances related to the development of bacteriophage-based vaccines, focusing on the vaccine delivery properties of this approach. Moreover, the immunological aspects of phage-based vaccines, as well as the applications of phage display for vaccine development, are explored. Finally, important challenges and the future of phage-bases vaccines is discussed.

## 2. Phage-Based Vaccines

The first study describing the use of phage particles as immunogenic delivery vehicles was reported by de la Cruz et al. in 1988 [12].

**Figure 1:**
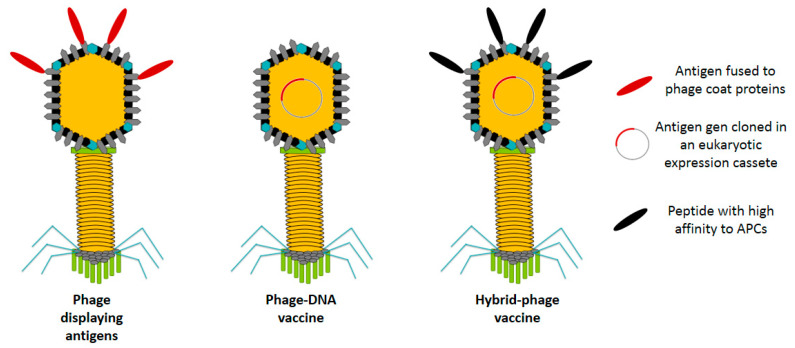
Simplified schematic representations of the three types of phage-based antigen delivery systems currently reported. An antigen delivery system based on a hybrid phage-DNA vaccine combines the concepts of the other two systems. It is based on a phage displaying on its surface peptides with specific affinity towards antigen presenting cells (APCs) and at the same time, it harbors a DNA plasmid encoding the therapeutic antigen in a eukaryotic expression cassette.

Nowadays, two main types of phage-based vaccines have been widely recognized: (1) phage display vaccines and (2) bacteriophage DNA vaccines [6,10]. The combination of these two strategies has resulted in the development of a third strategy, (3) the hybrid phage vaccine. Figure 1 shows the different phage-based approaches for antigen delivery.

### 2.1. Phage Display Vaccines

Phages are involved in a wide range of applications such as drug delivery, phage therapy, biosensors development and as vaccine delivery systems [6,13,14]. Many of these applications are possible as a result of the development of phage display technology, which lies on the manipulation of bacteriophages to present antigens on their surface. Until now, phage display vaccines have been used for preventing or treating several diseases including cancer, viral, parasitic and fungal infection as well as their use in immunocontraception and drug abuse, among others [2,15,16,17,18]. Vaccines targeting drug abuse consists on the use of phages particle displaying antibodies that block the effects of different drugs such as the cocaine [15].

#### 2.1.1. General Overview of Phage Display Technology

Bacteriophages are a specific class of viruses that infect and replicate into bacteria and archaea and are incompetent for eukaryotic infection [10,11]. These viral particles are made by genetic material, DNA or RNA, packaged in either simple or elaborate protein-made structures known as capsids. 

Phage display is a potent technology that consists on the expression of either peptides or proteins fused to coat proteins of the phage’s surface [11,19,20]. This technique has been used in biotechnology for several applications such as: directed evolution [21], identification of ligand binding sites (protein-protein, protein-ligand and protein-DNA interactions) [20], selection of monoclonal antibodies [22], protein-based drug discovery [23], development of epitope-based diagnostic tools, identification of B-cell epitopes [24] and for vaccine development [19,25]. Filamentous phages (M13, fd, and f1), lytic phages (T4 and T7) and the temperate phage lambda (λ) have been successfully used as display systems [10,19,20,26]. From all these systems, bacteriophage M13 is the most broadly used in phage display since its purification from an *E. coli* lysate is easier compared to other phages [10,27,28].

##### Filamentous Phages

Filamentous phages are a group of non-lytic viruses with a circular single-stranded DNA genome with a length around of 6.4 kb in the case of M13 phage [20,29]. As a result of the non-lytic nature of filamentous phages, these particles can be obtained in high titers with reduced bacterial contamination, making their purification easier and cheaper [30]. Due to their inherent viral particle features, filamentous phages are considered vaccine carriers with high immunogenic potential [11,31]. These particles are flexible protein-based cylinders composed of five coat proteins (pIII, pVI, pVII, pVIII, and pIX). From these, proteins pIII, pVI, pVII and pIX are the minor coat proteins, meanwhile pVIII is the major coat protein which surrounds the whole particle. pIII and pVIII proteins are the most used for the display of fusion peptides due to their specific location on the phage structure (Table 1) [10,32]. Short peptides (8 or fewer amino acids) can be effectively fused to many copies of the protein pVIII since the display level have been found to be dependent of the length and sequence of the peptide displayed [33]. Since the valency (protein copies per viral particle) has a direct relationship with the immune response to be generated, the proportion of immunogenic peptides displayed on protein pVIII has been extensively used for vaccine development [31,34,35]. On the other hand, protein pIII is preferred for the expression of larger peptides since the phage has fewer copies and steric effects may be negligible [28,36]. Furthermore, important advantages of using filamentous phages for the development of vaccines include that phages are stable under harsh conditions (pH and temperature), the phage size is determined by the length of the DNA molecule it harbors and the phage genome capability to be used as cloning vector [10,28,37]. Although these features surely encourage the use of filamentous phages as antigen display systems, a strategy to address their inefficiency to display peptides above 8 amino acids long on the major coat protein pVIII is needed to maximize the display flexibility of the system [38]. 

##### Lytic Phages

Lytic phages T4 and T7 have also been employed in phage display for vaccine applications [10]. The capsid proteins of phage T4, Hoc and Soc [39], can be used to display larger proteins at high copy numbers more efficiently than filamentous phages (Table 1) [40]. At the same time, T4 phage capsid proteins have demonstrated to promote an immune response in humans and mice [41]. In addition, oral administration of whole wild type T4 phage to humans demonstrated to be highly safe in clinical trials [39]. Some advantages of using T4 phages as vaccine carriers include the high immunogenic activity exerted by their capsid proteins, plasmid compatibility to allow engineering of dual protein expression and the absence of toxic proteins secreted. Based on these features, T4 phage has been preferred in some approaches as a display vector over filamentous phages commonly used [42]. 

On the other hand, the carboxyl-terminus of protein 10 B of phage T7 has been engineered to display heterologous peptides in antigen display strategies [43]. Some of the advantages of using the T7 phage for protein display include its high cloning capacity, high stability to harbor foreign gene inserts and its rapid propagation among other features [44]. In addition, humoral and cellular immune responses have been reported to be activated following the administration of recombinant T7 phage particles in animal models [45].

##### Temperate Phages

Although filamentous phages are the most common vectors used in phage display, the λ phage has also been proposed as an antigen display platform [46]. It has been reported that the λ phage can display properly folded antigenic peptides and 2–3 times larger fusion proteins than filamentous phages, offering a plausible alternative for complex antigens display [10,47]. For instance β-galactosidase, a protein larger than 400 kDa, have been properly displayed on the λ phage surface without affecting phage viability and morphology [35]. Moreover, it has been reported that the display of peptides using the λ phage generate particles with higher antigen densities when compared to the density of peptide displayed by filamentous phages [10,35].

In *vivo* and in *vitro* phage display systems.

In an in vivo phage display system, the expression and fusion of the displayed protein occurs at the phage infection stage. Although many large proteins have been successfully displayed in phage vectors using in vivo systems, limitations such as variations in intracellular expression, protein aggregation and deficient phage assembly have been described [48]. Such drawbacks can negatively impact vaccine development since the displayed proteins must be properly folded to exhibit the desired activity. On the other hand, in vitro display systems have demonstrated to overcome the limitations of in vivo display systems by promoting the correct fusion and further display of the protein of interest. This enhancement in protein expression and display efficiency in in vitro systems has been attributed to the use of purified components in a controlled environment [29].

#### 2.1.2. Phage Display in Vaccine Development

As mentioned in previous sections, phage display has been proposed as a potent tool for vaccine development [51,52]. This technology has been used for this purpose in two main strategies: (1) to produce vectors displaying antigens and (2) to identify new protective antigens [6]. The use of phage vectors as antigen display agents is focused on both, the development and production stages of a vaccine. On the other hand, phage display can be employed in early stages of the vaccine design process for the identification of novel antigens through the use of genetic engineering to generate random proteins and peptides aimed to interact specifically with a target, allowing the selection of the best binding antigen.

##### Phage Displaying Antigens

Phage display vaccines relies on the successful expression of antigens fused to phage surface proteins to produce viral particles with specific immunogenic activity [25]. Table 2 describes the phage display vaccines that have been currently reported. It is worth mentioning that anticancer vaccines successfully developed in recent years have already been used in cancer immunotherapy [53]. Administration of these anticancer phage vaccines stimulate the immune system of the host to produce antibodies against cancer antigens to reduce tumor cells proliferation [17]. To identify the best immunogenic antigen for phage-based cancer vaccines, several candidates have been evaluated. For instance, some antigens reported are: Epitopes from Epidermal Growth Factor Receptor (EGFR) [54], melanoma antigen gene (MAGE), and Fms-like tyrosine kinase 4 (Flt4) [55].

Since the discovery of the capacity of filamentous phages to exert anti-tumor activity in rabbits and mice [33], the use of phage particles as immunotherapy to treat melanoma tumors has become a very compelling research field. Studies on the administration of M13 and T4 phages to mice tumor models have demonstrated that the mechanism by which anti-tumor phages induce tumor elimination is mediated by the activation of tumor-associated macrophages in a Myeloid differentiation primary response 88 (MyD88)-dependent way [56]. Interestingly, the effect of phage administration was observed with a single dose of 1 × 10^11^ plaque forming units (pfu) injected subcutaneously. 

On the other hand, it has been reported that the administration of anti-tumor phages stimulates the infiltration of neutrophilic granulocytes, leading to the development of metastatic activity that results in damage of tumor tissue and reduction of viable tumor cells [57]. In other study, Ericksson et al. reported that the mechanism of action for the tumor degradation caused by bacteriophages is toll-like receptor (TLR) dependent. This study also reported effects on the production of proinflammatory cytokines, on the secretion of molecules necessary for antigen presentation by dendritic cells and co-stimulation of macrophages and T cells [57]. These findings suggest the high capacity of phage particles to induce immunogenicity and prompts the development of phage-based vaccines for cancer immunotherapy.

A phage-based vaccine aimed to treat amyloid plaque from Alzheimer disease produced an increased IgG response in a transgenic mice model, demonstrating the capacity of phage-based vaccines to activate humoral responses [16]. Immunocontraceptive vaccines have also gained attention for phage-based therapies. For instance, the Gonadotrophin Releasing Hormone (GnRH) antigen was displayed on the surface of T7 phages for immunocastration in a mice model [58]. Antiviral phage-based vaccines have also been successfully developed. Epitopes from HIV, hepatitis B, herpes simplex virus 1 (HSV-1), HSV-2 [34], Circovirus 2 (PCV2) [29], Human Respiratory Syncytial Virus [59] among others have been phage displayed.

##### Antigen Identification

**Figure 2:**
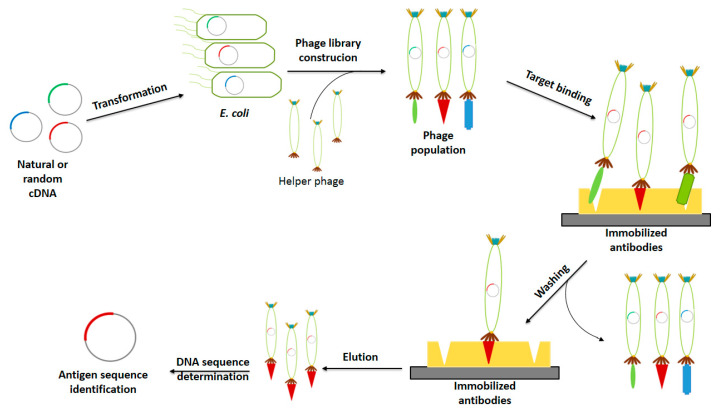
Antigen identification using phage display technology. The stages of phage display technology for antigen identification are phage library construction, phage selection and antigen identification. Adapted from [36].

Phage display has demonstrated to be an effective, inexpensive and fast technique for the identification of immunogenic proteins and peptides allowing the development of novel and more effective vaccines [19,36,64]. This methodology incorporates principles of genetic engineering and combinatorial chemistry [52]. It consists in cloning natural and random cDNA sequence variants into the phage genome to be expressed on the phage surface to generate a population of phages carrying different candidate antigens. From these libraries, phages with more affinity to the desired target (antibody for antigen identification) are selected and the DNA sequence encoding for the antigen is identified (Figure 2) [36]. The high efficiency and low costs that have been reported for the design of several vaccines using phage display technology for antigen identification are highly relevant features that must be taken into consideration while choosing the best platform for drug design [20].

This technology has allowed the identification of epitopes, mimotopes, bacteria adhesins and vaccine components (Table 3) [10,35]. One of the greatest advantages of using this method is the rapid identification and isolation of phages expressing specific targets [20]. At the same time, the protein pIII from M13 has been the preferred protein for fusion since high affinity of specific targets towards the displayed antigens have been observed [36].

Phage display technology has been applied for the identification of new and more specific linear and continuous antigenic determinant epitopes which normally have a length of 4–6 amino acids [20,69]. For instance, in a recent published paper, Chung-Tao et al. reported the use of phage display to identify novel enhanced epitopes from the envelope protein of dengue virus which was used later for the development of a DNA vaccine [24]. Authors concluded that the phage display methodology could be a key element for developing novel and more immunoprotective dengue vaccines [24]. Grabowska et al. demonstrated the applicability of phage display technology for the display and discovery of new protective virus HSV-2 epitopes [34]. Table 3 summarizes some of the epitopes identified using phage display technology. Altogether, this data supports the use of phage display for the development of new vaccines, diagnostic tools and immunotherapies [28]. 

Phage display libraries and biopanning have also been used for the identification of mimotopes of lipid and carbohydrate antigens which often present low immunogenicity [33,70]. Mimotopes are short peptides that structurally mimics epitopes, enabling a conformational interaction with an antibody [28]. These short amino acid sequences are used to produce antibodies against specific antigen epitopes and have several advantages over original antigens or epitopes since they are easy to produce for being short sequences, can mimic non-protein antigens, can be tested against undiscovered antigens and they have demonstrated to possess high bioactivity [17,70]. Thus, several mimotopes displayed on phages have produced effective in vivo responses in murine and swine models (Table 3). Nowadays, mimotope-based vaccination is considered an effective immunotherapy approach to induce specific antibody responses [71].

At the same time, use of phage display libraries have allowed the identification of amino acid sequences that mimics specific immunogenic and antigenic regions of toxins. By this approach, epitopes from botulinum neurotoxin A [72], the beta-mammal toxin Cn2 and Noxiustoxin (NTX) toxins have been discovered [73,74]. Recently, Jahdasani et al. used phage display technology for the identification of immunogenic epitopes from the scorpion venom of *Hemiscorpius lepturus* to develop a preventive strategy against scorpion bites [69]. Successful results were obtained demonstrating the applicability of this tool for the development of novel vaccine candidates in the antivenom area.

Phage display technology has also been used for the identification of new anti-parasite antigens. In this regard, antigens from *Brugia malayi* [75], *Plasmodium falciparum* [32], *Leishmania major* [23] have been elicited through this platform. By using phage display to identify peptides with binding capacity to the tegument of *Schistosoma japonicum*, Liu et al. discovered the ZL4 peptide which showed a potent antiparasitic activity [76]. Likewise, phage display for the identification of epitopes for *Taenia solium paramyosin* to develop a sole epitope-based vaccine has been described [77].

Nowadays, there are no vaccines against pathogens transmitted by ticks, which have been described as a serious health risk [65]. To overcome this problem, Becker et al. used oligopeptide phage display for the identification of antigens present in the saliva of the black-legged tick, *Ixodes scapularis*, which have been found to prevent pathogen transmission. Authors identified the metalloprotease 1 (MP1) as a potential candidate for the development of an anti-tick vaccine by testing MP1 immunogenicity in human sera [65].

As can be seen, phage display, a more than 30 years old technique, is an important tool for vaccine development and immunotherapy that had not been harnessed for these purposes until 10 years ago. At the same time, phage display has been applied for the enhancement of antigen-antibody interactions leading to the design of more potent vaccines [20]. The exploitation of this technique for vaccine development may lead to important advances for the prevention of several important infectious diseases [20,24].

### 2.2. Bacteriophage DNA Vaccines

DNA vaccination consists in the direct administration of a foreign DNA encoding an antigen to induce host immunity. For this approach, use of dendritic cells as transfection targets have demonstrated to increase immunity [35]. DNA vaccination has been proposed as a viable alternative when a protein or peptide vaccination is not viable or unsuccessful [3]. Several advantages of DNA vaccines have been described, for instance: antigen is folded correctly inside the host’s cell, downstream processing is not required which significantly decreases overall manufacture costs, null risk of vaccine to become pathogenic, DNA can be obtained in large quantities and high purity at relatively low costs and no strict storage conditions are required for DNA products [78,79]. Moreover, since this approach have demonstrated to stimulate both cellular and humoral immune responses, DNA vaccines have been proposed as promising immunotherapy treatments [80]. Unfortunately, standard DNA vaccines have shown poor immunogenicity in large primate trials, meanwhile in human trials the use of adjuvants such as dendritic cells have been identified as mandatory [80,81]. The low stability and the poor distribution of naked DNA vaccines diminish hugely the efficacy of the immune response, making imperative the use of a delivery vehicle [8]. To note, currently, there are not commercial DNA vaccines for human medical applications. However, novel vaccines based on nucleic acids have been rushed into clinical trials amid the SARS-CoV-2 pandemic, pointing out their promising efficiency as immune system stimulators [82]. In that regard, since phage particles have shown adjuvant properties, they have been proposed as new and suitable vehicles for DNA delivery.

In concept, a bacteriophage DNA vaccine is made of a eukaryotic expression cassette encoding a specific antigen controlled by target-specific promoters contained within a bacteriophage (Figure 1) [3,25]. The expression cassette must have the necessary regulatory sequences to allow the correct gene expression and protein folding of the antigen. In this strategy, phage particles serve as passive carriers by allowing the transference of the DNA encoded antigen to eukaryotic cells where the DNA will be expressed [6]. Phage-DNA vaccines are considered more advantageous than standard DNA vaccines since the former are more stable for storage, transport and administration (mainly via oral route), since DNA is protected from degradation by the capsid of phage particles. Although ʎ phage is the most common phage vector for DNA vaccination, filamentous phages have also been tested [37,83]. An important feature to consider of filamentous phage-DNA vaccines is the fact that phagemid vectors can efficiently contain multiple gene copies, which may allow immunization with various epitopes or antigens using a single delivery vector [37]. Table 4 describes the phage-DNA vaccines currently developed.

Furthermore, it has been demonstrated that phage-DNA vaccines can induce a more effective immunoprotection when compared to naked-DNA strategies [10,79]. For instance, March et al. reported a higher immune response by using a lambda-DNA vaccine when compared to the effect of a naked plasmid vaccine even at lower doses [79]. In other work, Clark et al. reported a higher immune response when using a DNA vaccine compared to the immune effect caused by the commercial vaccine Engerix B, based on a recombinant protein-small surface antigen (HBsAg) of hepatitis B. The enhanced performance of the DNA vaccine was attributed to a better folding of the protein expressed in the host, better compatibility with post-translational modifications on the antigen, the adjuvant effect of the phage particle, the presence of lipopolysaccharides and lipid incorporation when the protein is secreted [3].

Notably, phage DNA vaccines have exhibited more effectiveness when multiple doses (4 × 10^11^ phages per dose at intervals of 0, 5 and 10 weeks) are administered. This effect has been attributed to poor DNA expression in the first immunization since the host’s immune response focuses on phage coat proteins incapacitating a proportion of the DNA-carrying phages. However, the complete mechanism has not been elucidated [3]. Based on the favorable characteristics of phage particles and compared to other vaccine systems, production of phage DNA vaccines is simple and low-cost, the viral particle do not carry antibiotic resistance genes, the phage genome is suitable for the insertion of large DNA antigen sequences (up to 20 kb in lambda vectors) and are quite stable compared to standard DNA vaccines [3]. In addition, phage DNA vaccines are safer than DNA vaccines using adenovirus vectors since the bacteriophage is unable to replicate into eukaryotic cells [79].

### 2.3. Hybrid Bacteriophage Vaccines

Hybrid bacteriophage vaccines are produced by the combination of phage display and phage DNA vaccines. The most recent advances in phage-based vaccines have focused on improving the interaction and internalization of phage particles by antigen presenting cells (APC) to increase the immune response. This system relies on bacteriophages displaying proteins or peptides with high affinity to antigen-presenting cells or the antigen itself and at the same time they carry on their genome an eukaryotic expression cassette encoding a specific antigen with the final aim of increasing the immune response by combining both effects (Figure 1) [6]. For such reasons, this strategy has been proposed to be implemented as a directed therapy based on its capacity of delivering antigenic genes to activators of the immune response such as dendritic cells. It has been reported that this kind of vaccines are capable to successfully induce cellular and humoral immune responses [25]. Unfortunately, the mechanism for DNA delivery used by phages has not been completely elucidated [10]. Although the cellular internalization of phage particles seems to be quite efficient, the nuclear uptake efficiency must be improved. In 2008, Sartorius et al. developed a double-hybrid filamentous bacteriophage fd co-displaying peptides recognized by the Major Histocompatibility Complex (MHC) class I and MHC class II cell surface receptors and epitopes from the antigen MAGE aiming to enhance the anti-tumor immune activity based on CTL responses [62]. By observing an increase in the CTL response through the administration of hybrid phages, Sartorius et al. proposed this strategy as a potent tool for the development of more effective anti-cancer vaccines.

## 3. Pros and Cons of Bacteriophage-Based Vaccines

Ideally, a vaccine carrier should be easy to produce in large quantities, safe and capable to effectively present antigens to the immune system to induce a proper immune response. Viral particles and more specifically bacteriophages have demonstrated to possess many of the properties previously mentioned. Thus, in recent years bacteriophage-based vaccines have gained attention for medical applications considering their low production costs, relative simplicity to prepare and to genetically modify, easiness to produce at large scales, their adjuvant capacity, high stability under a wide range of pH and high temperature, resistance to nucleolytic and proteolytic enzymes and desiccation [25,29,47].

The capability of phages to be genetically modified to be used as display or delivery systems represents an important aide in emergency situations, for instance outbreaks of infectious pathogens [35]. In this context, recombinant phages can be produced in large amounts at low cost by basic methods making phage-based vaccines more cost-effective than standard or classical vaccine development strategies [59]. According to Munira et al. the cost of producing recombinant vaccines is estimated on average of 2 USD per dose [84]. On the other hand, in, Torres-Acosta et al. estimated the production cost for a phage-based vaccine was around $0.9 USD per dose (10^12^ pfu) [85]. These estimations suggest a 55% reduction on the production costs of phage-based vaccines compared to recombinant-based vaccines, supporting the implementation of phages in vaccine development strategies.

The high stability exhibited by filamentous phage preparations and their capacity to protect antigens from degradation makes bacteriophages ideal vaccine vehicles based on their storage and transportation resilience and delivery features. Based on the discussion in previous sections of this review, phage-based vaccines are more suitable for immunization than DNA naked or recombinant vaccines. In that regard, Brigati and Petrenko evaluated the stability of landscape phages—multivalent pVIII-type filamentous-phage constructs with unique structures and properties—expressing peptides fused to protein pVIII at high-temperature (63 °C) and observed that these particles retained stability without affecting their binding sites [86]. This capability of phages is quite remarkable and has to be taken into consideration for the development of vaccines that will be distributed to remote areas where no adequate storage conditions are found or for veterinary applications that requires vaccine administration in the native habitats of animals [11].

The use of phages as antigen carriers has the advantage of increasing the half-life of the antigen in the blood stream facilitating the activation of T-helper cells, enhancing the immune response [87]. To study the immunogenicity efficiency of phage display, Wu et al. compared the immune response of recombinant antigens with phage-displayed antigens and reported that the antigens expressed on the phage surface exhibited a better refolding leading to a more efficient activation of the immune system [49]. This results, along with the increase observed on cellular immunity by the administration of hybrid-phages displaying APCs-targeted peptides [11], demonstrates the remarkable properties of phage-displayed antigens

Bacteriophages are the most abundant organisms that inhabit the entire planet [88], suggesting their high capability to coexist with a plethora of micro and macro organisms. For such reason, it is not unusual that these particles have proved to be harmless in mammalian models even in oral administration trials in humans approved by the FDA [10,29,39,89]. To reinforce even more the safety of phage administration, as any other viral particle used as vaccine, bacteriophages can be physically or genetically inactivated as well [90]. Furthermore, phage particles have also been used in therapeutic applications in humans without side effects being observed [91]. Thus, the safe application of phage-based vaccines have been demonstrated as bacteriophages are not capable to infect and replicate into eukaryotic cells and no side effects have been reported contrary to the outcomes observed in mammalian model using other viral vectors [10].

Although many proteins and peptides have been successfully displayed in bacteriophages, the correct display of molecules on the phage surface is still considered a potential drawback for the development of these vaccines. The issue relies on two related subjects; proper folding of the polypeptide sequences displayed, and the epitope density displayed. Both features, correct folding and presence of enough active epitopes to generate a significative immune response have a direct effect on the overall efficiency of these vaccine [25]. An equally important factor to have in mind while designing any kind of vaccine is the administration route. Although phage particles have showed to be safe for animal and human use, phages administered by oral route may infect gut bacteria, potentially leading to a dysbiosis. The infection and further replication of phages could induce the release of endotoxins from infected bacteria, increasing the risk of host damage [92]. This risk can be eliminated by using non-lytic bacteriophages such as the M13 phage.

## 4. Immunological Basis of Phage-Based Vaccines

Several authors have described that the natural immunogenic properties of phage particles relies mainly on their chemical structure (phage proteins and viral DNA) [47]. This capacity makes bacteriophages excellent candidates for their use as antigen delivery vectors. In recent years, although not completely, the immunogenic effects in hosts driven by phage-based vaccines have been described, allowing a more rational design of novel and better phage vaccines. 

### 4.1. Immunogenic Properties of Phages

Several authors have reported the capacity of filamentous phages to act as natural vaccine adjuvants since these viral particles are capable to achieve an effective antigen presentation to immune cells [47]. This notable feature allows phage-based vaccines to enhance the stimulation of the immune response, obtaining better results than conventional vaccines even at relatively low doses [52,93]. In addition, it has been reported that phage-based vaccines produce less variability between subjects than conventional vaccines [25]. It has been suggested that filamentous phages evolved to induce low non-specific immune response in eukaryotic organisms [94]. However, filamentous phages still can induce an effective activation of the immune machinery by themselves apart from activation of both cellular and humoral immune responses by being engineered to directly interact with APCs, giving them an advantage over other delivery systems [29]. 

The immunogenicity exhibited by filamentous bacteriophages have been demonstrated to be caused not only by their repeating coat proteins but also mediated by the phage DNA itself [57,95]. This was demonstrated by the administration of DNA from M13 phage to mice in a strategy to induce in vivo heterologous expression of interferon (predominantly the beta type) to confer the mice protection from a vaccinia virus infection. The immune response was in part attributed to the presence of CpG motifs in the phage genome [3,96]. Phage vaccination trials in mice have demonstrated that CpG motifs stimulates T helper type cells (Th1) and B cells leading to an enhanced immune response [97,98,99]. CpG motifs have been also described to be involved in the stimulation of the Toll-like receptor 9 (TLR9) signaling cascade, modifying the production of different immunoglobulin classes [100]. In the case of the T4 phage, the major capsid protein gp23 and the highly immunogenic outer capsid protein (Hoc) have shown high antigenicity [55]. Hashiguchi et al. investigated the immune response derived from the administration of M13 phages in an in vivo murine model. Authors found that the administration of phages induced an equivalent immune response as the typical immunogen Sheep Red Blood Cells (SRBC), reinforcing the theory that M13 phage may be an important vector for vaccine delivery [95]. In another report, it was found that filamentous phages induce a stronger immune response than the carrier protein ovalbumin [93], demonstrating that bacteriophages can induce an immune responses by their own, supporting their use as promising vaccines carriers.

Interestingly, van Houten et al. reported that reducing the complexity-quantity of different B-cell epitopes of immunodominant epitopes of filamentous coat proteins in phages enhanced the antibody response against synthetic peptides fused to the phage surface [31]. Thus, the adjuvant-like effect exhibited by phages in various vaccination studies along with their capacity to effectively present peptides or proteins to the immune system leading to the activation of cellular and humoral immune responses [40] demonstrates that engineered phage particles are a proper strategy to enhance the efficacy and safety of viral particle-based vaccines.

### 4.2. Immunological Mechanism of Phage-Based Vaccines

**Figure 3:**
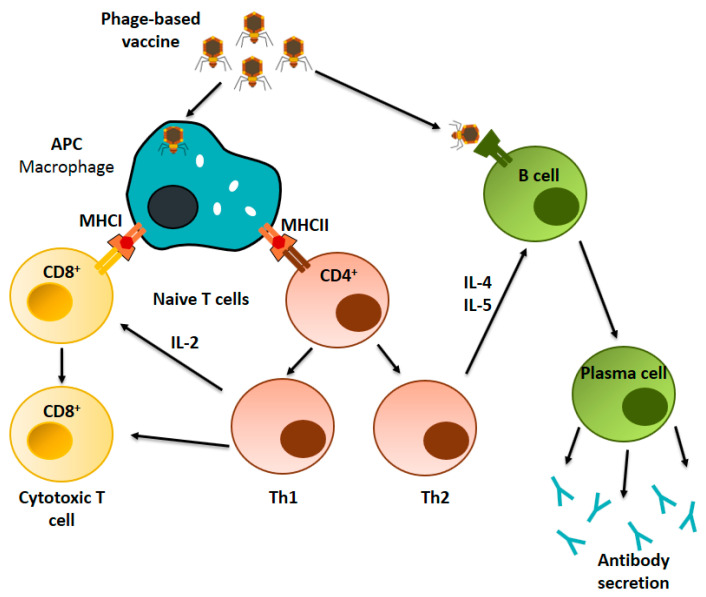
Immunological mechanism of phage-based vaccines. Phage-based vaccines can stimulate both humoral and cellular responses. Phage particles displaying-antigens are taken up by APCs. The antigen is then processed and presented on MHC-I and MHC-II molecules which leads to the activation of T cells. The direct recognition of the phage particle happens simultaneously, promoting the generation of plasma cells and further secretion of antibodies.

From a metabolic point of view, bacteriophages are considered inert antigen particles whose cell internalization mechanisms are still being studied. It is widely accepted that phages are internalized by endocytosis and processed by APCs [101]. Once phage particles are internalized by APCs, antigens are processed and presented through the major histocompatibility complex (MHC) class I and II pathways (Figure 3), stimulating cellular and humoral immune responses [52]. Phage-displayed antigens have been described to induce specific CD4+ and CD8+ T cells lymphocytes (CTLs) responses, leading to the production of specific antibodies and activation of T helper cells without the utilization of supplementary adjuvants. Also, it has been reported that macrophages are capable to internalize phages via phagocytosis [102].

#### 4.2.1. Cellular Immune Response 

Antigen presentation via the MHC class I pathway have been demonstrated to activate the CTL through the interaction with CD8+ T cells (Figure 3). It has been suggested that standard vaccines (soluble exogenous antigen or inactivated pathogens) fail to stimulate the MHC class I pathway, and therefore are incapable to activate T cells, which are important to promote an optimal immune response [103]. In that sense, it has been reported that filamentous phages displaying antigens are efficiently processed and presented by the MHC class I and class II pathways, demonstrating their high potential as positive stimulants of the immune system [37,60,62]. Activation of the MHC class I pathway and further triggering of CTL responses have been reported to play a key role in anti-cancer and anti-viral drug approaches [25]. In that regard, Wan et al. reported that filamentous phages displaying an exogenous antigen successfully triggered a MHC class I-restricted CD8+ T cell response [61]. In another work, Sartorius et al. demonstrated the capacity of hybrid filamentous phages to induce T cell-dependent CTL responses when specific epitopes were displayed on the phage surface [62]. Moreover, phages can stimulate APCs for the secretion of costimulatory molecules required for T cell activation [57].

Phage display technology allows the design of engineered phages capable to express molecules that specifically targets presenting cells for the induction of a strong cellular response [104]. Iwagami et al. reported T cell activation of CD4+ and CD8+ with the co-expression of CD154 and CD137 markers after administration of a ʎ phage-based vaccine displaying the peptidic antigen Aspartate-β-hydroxylase (ASPH) [47]. In the same study, the response also included an increase on the secretion of IFNγ and the ASPH antigen displayed in the ʎ phage generated specific CD4+ and CD8+ responses in vitro [47]. Thus, phages capacity to induce a CTL response supports the use of bacteriophages as vaccine carriers capable to enhance the immune response produced in stand-alone antigen strategies. Likewise coat proteins, bacteriophage DNA has exhibited to possess an immunostimulatory effect. In that regard, Cuesta et al. reported that a DNA fragment of the domain I of the coat protein III of filamentous phages is capable to enhance the T helper 1 humoral and cellular immune responses when fused to a single-chain variable fragment (scFv) [105]. 

Antigen presentation to naïve CD4+ T cells via MHC-II pathway has demonstrated to leads to the activation of Th1 and Th2 cells (Figure 3) [94]. Also, in vitro and in vivo studies have confirmed that recombinant filamentous phages are capable to trigger a strong immune response mediated by T helper cells. This response is mediated by phage components such as the coat proteins, phage DNA as well as phage-associated lipopolysaccharide (LPS) which can direct the activation of Th1 and Th2 cells [11,34]. To study the effects of phages in the immune system, Iwagami et al. immunized mice using a ʎ phage vaccine at a dose of 10^10^ pfu and observed an increased secretion of specific cytokines of Th1 (IFN-γ and TNF-α) and Th2 (IL-4, IL-6 and IL-10) [47]. In the same study, authors observed that activation of Th1 generated a CTL response, suggesting that phage particles may be great candidates to consider for the development of vaccines focused on CTL response activation. On the other hand, a phage-mimotope vaccine against *Fasciola hepatica* showed a mixed Th1 and Th2 cells responses, advocating the approach of using phage particles in vaccination strategies targeting cellular responses [106].

#### 4.2.2. Humoral Immune Response

Phage particles have demonstrated their capacity to induce humoral responses through the direct activation of B cells as well as through the activation of Th2 cells (Figure 3) [94]. It has been proposed that for a better B cell response, antigens must be presented in a repetitive and organized configuration [43]. Studies focused on the administration of phage display vaccines have also shown the induction of immune response in animal models injected with recombinant vaccines and mimotopes expressed and fused to coat proteins of phages at a dose of 10^11^ pfu [95]. Also, it has been reported that the antibody response induced by M13 phages is limited to recognition of the first 12 N-terminal residues of protein pVIII and to the external domains of protein pIII [95]. In 2010, Hashiguchi et al. studied the in vivo immune response induced by M13 phage with the aim to characterize this effect for vaccine development [95]. The researchers administered a solution containing highly purified M13 phages (10^11^ pfu) to mice and observed the antibody response. They reported a strong primary response based on high production of IgG antibodies which was attributed to a complete dependence on the activation of the MyD88 pathway involved in innate immune response [95]. Other studies have suggested that bacteriophages may play an important role in the innate response activation by stimulation of tumor-associated macrophages (TAMs), which secretes immunomodulators that assists the recruitment of neutrophils [57]. Unfortunately, the complete molecular mechanism of the activation of innate immunity by phages has not been completely elucidated. 

In 2012, Thomas et al. produced a divalent subunit vaccine expressing Green Fluorescent Protein (GFP) as model antigen and the HIV-1 TaT protein to enhance cell uptake and thus establish a subunit λ-based vaccine [35]. This platform allows the display of many epitopes at the same time, providing an easy and rapid approach to develop novel vaccines against emerging pathogens. Authors reported a strong immune stimulation of splenocytes using the λ-based vaccine demonstrating the effectiveness of phage preparations to induce a strong adaptive immune response. Cytokine profile was also evaluated, and the results showed a high IFNγ secretion by splenocytes, suggesting a Th1 activation which lead to higher IgG2a isotype production. Furthermore, it was observed that host’s antibodies against the phage-based vaccine recognized more epitopes than those produced with naked DNA vaccines. 

## 5. Administration Routes for Phage-Based Vaccines

For long time, subcutaneous and intramuscular injections have been acknowledged as the most common routes for vaccine administration. Wild-type and recombinant M13 phage particles have proved their capacity to cross the gastrointestinal barrier and remain stable, suggesting the use of phage nanoparticles as antigen delivery systems in oral route administration strategies [107,108]. Nevertheless, based on their native capacity to recognize and infect bacteria, it has been reasonably speculated that oral administration of phages could cause an unbalance of the gut bacteria, compromising the host’s health [52]. Fortunately, diverse strategies focused on improving the safety of phage-based vaccines administered by of oral route have been developed [109]. A promising strategy could be the utilization of non-lytic phages, as the M13, to diminish the risk of damaging the host’s gut microbiome since these phages are not suited to destroy bacterial cells. Another strategy is based on the use of viral particles with non-functional tail fibers, required for target recognition and further infection, to make them unable to infect bacterial cells without reducing their capacity to act as vaccine delivery systems [25]. Moreover, oral administration of phage-based vaccines has demonstrated strong immunostimulatory effects, reinforcing the idea of using phage particles as oral vaccine carriers [110,111].

## 6. Final Remarks and Challenges

Despite the considerable amount of evidence supporting the use of bacteriophages as vaccines, their clinical application still requires further research and support. Fortunately, U.S. Food and Drug Administration (FDA) has approved recently the first clinical trial for the use of phage therapy in humans. This phage therapy trial consists on the utilization of a bacteriophage combination to treat a resistant *Staphylococcus aureus* infection. This step opens the opportunity to develop more effective clinical trials involving the use of phage particles for vaccine development. 

The current available knowledge describing the biology, physiology, and purification of phage particles should help to design adequate clinical trials. However, it is important to note that these clinical evaluations are quite expensive which limit their application to phage-based products. Moreover, the wide diversity of phage-based vaccines could help in the development of combinatorial vaccination strategies that may surpass issues of current vaccination strategies. Based on the wide range of applications that phage-based bioproducts may have and taking into consideration the effectiveness demonstrated of phage-based vaccines currently developed, the design of clinical trials should be taken as a priority for approval of these bioproducts [6]. Phage-based vaccines made without the insertion of foreign DNA (in vitro display) into the phage genome can be considered as natural bioproducts since no risk of genetic transfer exists. These considerations should be acknowledged to accelerate the commercial use of phage-based vaccines.

Phage-based vaccines represent a promising approach for vaccine development since this approach offer important advantages over standard vaccine delivery systems. Further studies are required for a better understanding of the immunological mechanism of phage vaccines in order to develop more specific antigen delivery systems. Furthermore, immunization protocols aimed to test the effect of higher phage vaccine doses as those currently studied (10^10^ and 10^11^ pfu) should be implemented. Future research of phage-based vaccines will focus on optimization the immunogenicity of the antigens displayed. Finally, it has been suggested that the expression of mimotopes in a tandem configuration and fused to the pVIII protein of bacteriophages could induce an enhanced effect on the immune response compared to the effect observed in other vaccination strategies. Therefore, this approach should be thoroughly investigated to improve the efficacy of phage-based vaccine.

## Figures and Tables

**Table 1:** Phage vectors used for vaccine delivery and their main features for antigen display. Copy number is the number of antigens displayed per viral particle.

Schematic Representation of Phage Display Vectors	Proteins Used for Fusion Display	Copy Number	Size of Displayed Antigen	References
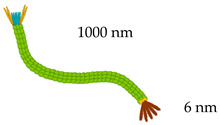 **Filamentous (M13, fd and f1)**	pVIII (major coat protein 5 kDa) 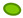	2700	6–8 aa	[36,48]
	pIII (minor coat protein 45 kDa) 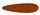	4–5	Large proteins	
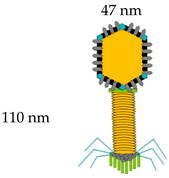 **T4**	Soc (small outer capsid 9 kDa) 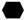	810–960	Up to 840 aa	[49]
	Hoc (highly antigenic outer capsid 40 kDa) 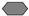	155–160	Up to 183 aa	
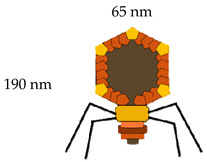 **T7**	gp 10 A 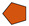	415	40–50 aa	[6,43]
	gp 10 B	1	Up to 1200 aa	
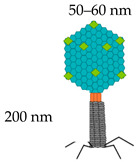 **Lambda**	gpD (outer capsid protein) 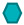	420	Up to 1024 aa	[29,50]
	gpV (tail protein) 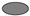	192		

**Table 2:** Design, characteristics, use and main findings after administration of current bacteriophage-based vaccines.

Vaccine Type/Antigen Displayed	Model of Study	Main Effects of Phage-Based Vaccine Administration and Comments	Dose	Phage Used	Phagemid Vector	Protein Used for Phage Display	*E. Coli* Strain Used for Phage Assembly/Production	Reference
**Anti-cancer** Melanoma Epitope MAGE-A1_161–169_	Mice and cell lines YAC-1 and B16.F10	Protection from tumor growth Specific cytotoxic T cell (CTL) responses, increased NK activity, CD 8+ response and Delayed-type hypersensitivity	50 ug phage particles i.p	fd	pfd8wf	pVIII	TG1	[60]
**Anti-viral** Hepatitis B virus epitope S_28–39_	Female BALB/c mice	MHC I restricted HBs specific CTL response	10 ug i.d i.p	M13	pC89	pVIII	XL1-blue	[61]
**Anti-viral** HRSV G glycoprotein-epitope _173–187_	BALB/c mice	Induction/stimulation/promotion of strong immune response of animals against RSV infection	1 mg i.p	fd	Bacteriophage vector Fuse 5	pIII	K91 Kan	[59]
**Anti-parasite** Peptides GK1, KETc12, KETc1 and KETc7 *Taenia solium* cysticercosis	Pig	Reduction of 70% of tongue Cysticercosis, 54% of muscle-cysticercosis and 87% of total cysticerci Similar efficacy to the synthetic vaccine	2 mL, 10^12^ phage particles of each s.c	M13	M13KE	pVIII	TG-1	[2]
**Anti-liver cancer** ASPH peptides	Murine	Reduced Hepatocellular carcinoma growth and progression. Increase in CD4+ and CD8+ response Specific Th1 and Th2 cytokine secretion.	10^10^ pfu s.c	λ	pVCDcDL1A	gpD	n.r	[47]
**Anti-viral** Epitopes of the glycoprotein G of HSV-2 (gG2)	Balb/c mice	Promotion of protective immunity in a live challenge model of HSV-2 infection	n.r.	Fd-tet	n.r.	pVIII	K91Kan	[34]
**Anti-cancer** EGFR gene of extracellular domain of chicken xenogeneic EGFR	Male Kunming mice	Induction of specific anti-EGFR antibodies (Humoral immune response) Reduction of tumor progression immunity against EGFR-positive tumor.	2.5 × 10^13^ pfu	T7	n.r	10 B	n.r	[54]
**Anti-cancer** EGFR ICR-62 binding peptide mimotope	BALB/c female mice	Humoral immunity induced in mice Reduced tumor growth in ectopic Lewis lung carcinoma animal model *In vivo*: no response	10^12^ pfu s.c	M13	pAK8-GVO	pVIII	TG-1	[17]
**Contraceptive** 3 Gonadotrophin Releasing Hormone (GnRH) fragment 43 kDa	Male BALB/c mice	Specific anti-GnRH antibody faster than conventional vaccine Spermatogenesis suppression	10^10^ pfu s.c	T7	n.r	10 B	BL21	[58]
**Anti-viral** Ectodomain of influenza A virus channel protein M2 (M2e)	Female BALB/c mice	M2e-specific serum antibody responses T cell response Reduced viral load. Protection against Influenza A virus	10^9^ pfu s.c	T7	n.r.	10 B capsid protein	BL21	[43]
**Anti-cancer** Mouse Fms-like tyrosine kinase 4 (Flt4)	Mice	Induction of anti-Flt4 antibody Induction of antitumor immunity Inhibition of metastasis in Lewis lung carcinoma	10^11^ pfu s.c	T4	T4-Z	Soc	BL21	[55]
**Hybrid Anti-cancer** HLA-DR-restricted Th cell peptide epitope p23 and MAGE-A10_254–262_ or p23 and MAGE-A3_271–279_ from the HIV-1-RT	Human cell system in vitro humanized murine model in vivo	Induction of specific and potent CTL response Hampered tumor growth	140 µg phage particles s.c	fd	pTfd8p-66 for p23 peptide	pVIII	TG1 rec O	[62]
**Anti-cancer** Vascular endothelial growth factor receptor 2 (VEGFR2)	Mouse tumor model Male C57BL/6J	Induced anti-tumor immunity Induction of anti-angiogenesis activity Induction of CD4+ T cell–mediated effector mechanisms	5 × 10^11^ pfu s.c	T4-S-GPDS	pD-mVEGFR2	Soc C-terminus fusion	BL21 or HB101	[40]
**Dual display** of swine fever virus (CSFV) major antigenic determinant cluster mE2 and CSFV primary antigen E2	Female BALB/c mice	Enhanced immune response High antibody titers	10^10^ pfu s.c	T4-Zh^−^	pcDSW	Soc C-terminus fusion and Hoc N-terminus	BL21	[49]
**Anti-bacteria in vitro display** Protective antigen (PA) from *B. anthracis*	Female CBA/J mice	Production of immunogenic particles High antibody titers Neutralization of anthrax lethal toxin	10^10^ pfu i.m	T4 Hoc-soc-	pET-15b	Hoc N-terminus	P301 (sup-)	[48]
***Yersinia pestis* (Plague)** capsular F1 and calcium response V antigen	Female BALB/c mice	Complete protection against pneumonic plague TH1 and TH2 responses	10 ug phage particles i.m	T4 Hoc-soc-	pET-28b	Soc	BL21	[63]

HRSV: Human Respiratory Syncitial Virus; i.d: Intradermal; i.p: intraperitoneal; s.c: subcutaneous; pfu: Plaque forming units; MHC: major histocompatibility complex; ASPH: Aspartate β-hydroxylase; n.r.: not reported; Herpes Simplex Virus (HSV). EGFR: Epidermal Growth Factor Receptor; F: Filamentous. i.m: intramuscular. HLA-DR: Human Leukocyte Antigen-DR isotype; RT: Reverse Transcriptase; T4-S-GPDS: T4 bacteriophage nanoparticle surface gene-protein display system.

**Table 3:** Cases of epitope, mimotope and antigens identification through phage display describing the main findings in regards of the phage display protocol used for selection and the prophylactic and therapeutic results reported.

Antigen Source	Antigen Identified	Phage/Phagemid Vector	Screening Antibodies	Prophylactic and Therapeutic Effects	Reference
Ixodes scapularis ticks salivary gland	Metalloprotease (MP1)	pHORF3 M13	3 biopanning rounds with human serum antibodies against salivary gland homogenate (SGH)	No evaluated	[65]
EGFR gene	EGFR mimotope Triple tandem repeat	PAK-8 M13 pVIII	Anti EGFR monoclonal antibody ICR62	Lewis lung carcinoma tumor model	[33]
				Reduced tumor growth	
				High level of cytokines Raised	
				humoral response	
				Anti-cancer activity	
*Leishmania infantum (syn. L. chagasi)*	Peptide 5	M13	Polyclonal IgGs from *L. infantum* infected dogs	High immunogenicity	[7]
				Protective effect vs. *L. infantum* in mice model	
*Mycobacterium leprae*	Anti *M.leprae* epitopes	M13 (pIII)	Human antiserum	High immunogenicity in mice	[66]
				High antibody titer	
Salmonella Typhimurium	Novel immunogenic antigens	pHORF3 M13	Serum from infected pigs	High immunogenicity	[67]
Tetanus toxoid (TT)	Novel binding peptide anti TT	pGEX-4T_1_ M13 (pIII)	F13 Fab from human	No evaluated	[68]
*Trichinella spirallis*	Peptide 8F7	M13 (pIII)	mAb 8F12	Larval reduction in vaccinated mice	[18]
				High levels of IgG1	

**Table 4:** Description of the antigen, model of study, main findings, design features and administration of antiviral bacteriophage DNA vaccines.

Vaccine	Model of Study	Main Effects	Doses	Phage Used	DNA Cloning Vector	Promoter	Reference
**Anti-viral** Hepatitis B Surface antigen (HB)	Mice and rabbits	Anti HB response	Mice: 5 × 10^9^ pfu (250 ng of DNA)	λ	λ-gt11	CMV	[79]
			Rabbits: 4 × 10^10^ HBsAg pfu (2 ug of DNA)				
**Anti-viral** Small surface antigen (HBsAg) of hepatitis B	Rabbits	Strong antibody response (IgG, IgM)	4 × 10^10^ pfu	λ	pRcCMVHBs(S) λ-gt11	n.r.	[3]
**Anti-viral** Herpes simplex virus 1 (HSV-1) glycoprotein D	Mice	Anti-HSV-1 neutralizing antibodies Dose-response effect CTL response	1.4 × 10^13^ pfu (50 ug DNA)	M13	pcDNA3-gD plasmid	Human cytomegalovirus immediate-early	[37]
**Anti-viral** Human papillomavirus (HPV)-16 E7	C57BL/6 mice	Reduction of tumor volume	2 × 10^12^ particles	λ ZAP	n.r.	CMV	[46]

CMV: Cytomegalovirus; pfu: Plaque forming units, n.r.: not reported.
